# Should Endovascular Repair Be Reimbursed for Low Risk Abdominal Aortic Aneurysm Patients? Evidence from Ontario, Canada

**DOI:** 10.1155/2011/308685

**Published:** 2011-06-24

**Authors:** Jean-Eric Tarride, Gord Blackhouse, Guy De Rose, James M. Bowen, Hamid Reza Nakhai-Pour, Daria O'Reilly, Feng Xie, Teresa Novick, Robert Hopkins, Ron Goeree

**Affiliations:** ^1^Programs for Assessment of Technology in Health (PATH) Research Institute, St. Joseph's Healthcare Hamilton and Department of Clinical Epidemiology & Biostatistics, Faculty of Health Sciences, McMaster University, Hamilton, ON, Canada L8P 1H1; ^2^Division of Vascular Surgery, Department of Surgery, London Health Sciences Centre (LHSC), London, ON, Canada; ^3^Division of Vascular Surgery, Department of Surgery, Faculty of Medicine, University of Western Ontario, London, ON, Canada

## Abstract

*Background*. This paper presents unpublished clinical and economic data associated with open surgical repair (OSR) in low risk (LR) patients and how it compares with EVAR and OSR in high risk (HR) patients with an AAA > 5.5 cm. *Design*. Data from a 1-year prospective observational study was used to compare EVAR in HR patients versus OSR in HR and LR patients. *Results*. Between 2003 and 2005, 140 patients were treated with EVAR and 195 with OSR (HR: 52; LR: 143). The 1-year mortality rate with EVAR was statistically lower than HR OSR patients and comparable to LR OSR patients. One-year health-related quality of life was lower in the EVAR patients compared to OSR patients. EVAR was cost-effective compared to OSR HR but not when compared to OSR LR patients. *Conclusions*. Despite a similar clinical effectiveness, these results suggest that, at the current price, EVAR is more expensive than open repair for low risk patients.

## 1. Introduction

In 2002, the Ministry of Health and Long Term Care (MOHLTC) of Ontario conducted a review of primary studies on endovascular repair (EVAR) for abdominal aortic aneurysms (AAA) as well as a review of previous international and Canadian Health Technology Assessments (HTA) [1]. Due to the informational uncertainty associated with the long-term effectiveness of EVAR, the MOHLTC recommended that an Ontario-specific evaluation of the technology was warranted. To evaluate EVAR, the MOHLTC provided funding on a one-time basis to support a 24-month EVAR evaluation at London Health Science Centre (LHSC) Endovascular Program [1]. A condition of this funding was that LHSC would collaborate with the Programs for Assessment and Technology in Health (PATH) Research Institute at St. Joseph's Healthcare Hamilton to design and conduct a “field evaluation” to support the assessment of the effectiveness and cost-effectiveness of EVAR compared to open surgical repair (OSR) in Ontario. Following the evidence generated by this study, the Ontario Health Technology Advisory Committee (OHTAC) recommended increased access to EVAR in high risk patients [2]. As a result, EVAR was changed from an uninsured to insured provincial service (i.e., a fee code introduced) and several vascular programs in the province were restructured to accommodate EVAR for high risk patients. This funding decision was also consistent with the 2005 recommendations of the Canadian Society for Vascular Surgery (CSVS): “EVAR should be the procedure of choice for patients with suitable vascular anatomy who are at intermediate or high risk (6%–10%) for perioperative morbidity or death with open repair. For patients at low risk (2%–4%), open repair remains the current standard …” [3]. Following an updated review of the evidence in 2010, OHTAC did not recommend the use of EVAR in low risk patients [4].

In contrast, in the UK, the National Institute for Health and Clinical Excellence (NICE) recently recommended EVAR for all patients suitable for surgical intervention (i.e., OSR) independently of their surgical risk [5, 6]. Although NICE economic model supported that EVAR was not as cost effective in patients with a good “fitness” (e.g., low surgical risk), NICE estimated that it was not possible to exclude a category of patients (e.g., low risk) due to the lack of a standardized definition of fitness. In both Canada and the UK, it is recommended that discussions take place between clinicians and patients and the centers of excellence for EVAR be created. However, the decision to reimburse EVAR in Ontario was based on a one-year prospective study in which patients were stratified to the different interventions (OSR or EVAR) as a function of their baseline surgical risk according to scoring algorithms [7]. In particular, in our study, EVAR was not offered to patients at a low risk of surgical complications. Since the review by Jonk of cost-effectiveness of AAA repair and the publication of this trial-based cost-effectiveness study in high risk patients [8, 9], recent studies have offered mixed results when EVAR is compared to OSR [10–15]. However, no studies have to this point presented clinical, health-related quality of life (HRQoL), and economic data based on risk classification. To better inform decision-making regarding the diffusion of EVAR, this paper presents unpublished data on the one-year clinical, HRQoL and economic outcomes associated with OSR in low risk patients and how they compare with EVAR and OSR in high risk patients. We also report 5-year mortality data on EVAR patients. 

## 2. Methods

### 2.1. Study Design and Participants

All patients requiring elective repair of their AAA (AAA > 5.5 cm) at LHSC, Ontario, in 2003–2005 were invited to participate in a prospective observational study of EVAR against OSR for patients at a high or low surgical risk for perioperative death and morbidity. Demographic, medical, HRQoL, and resource utilization data was collected from participating patients at baseline, discharge time from hospital, and at one, three, six, nine, and twelve months postsurgery. The details of the study design and methods have been previously published but are briefly presented below [8].

### 2.2. Treatment Algorithm

**Figure 1:**
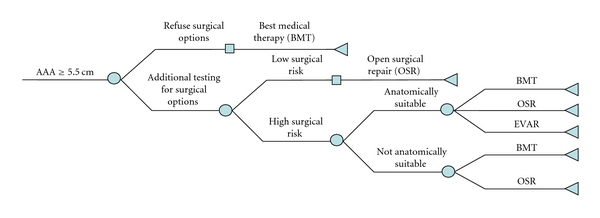
Treatment algorithm for elective repair.

The method of AAA repair was determined following institutional clinical criteria for endovascular aneurysm surgery and through discussion with the patient [3]. The eligibility of patients for surgical options was evaluated based on the presence of comorbidities [16]. Surgical repair of AAAs in patients considered to be of low surgical risk was completed with OSR. For patients anatomically suitable for EVAR, the choice of EVAR or OSR was presented as surgical options. High risk patients not anatomically suitable for EVAR were treated using OSR. All patients were offered best medical treatment should they not chose surgical options.  Figure 1 presents the treatment algorithm. In particular, all EVAR patients were at high surgical risk.

### 2.3. Clinical Outcome Measures

Several measures of clinical outcomes were used in this study including primary technical success (PTS), intraoperative and postoperative complications, thirty-day and 1-year mortality, as well as overall survival. Five-year mortality rates and causes of death were also presented for EVAR patients as these patients are followed up annually. Three definitions had to be met all together for primary technical success for EVAR: (1) successful introduction and deployment of the device, (2) absence of surgical conversion or mortality, and (3) absence of type I or III endoleaks, or graft limb obstruction [17]. For OSR patients, PTS required the successful replacement or bypass of the aneurysmal segment with a prosthetic graft in the absence of mortality or graft thrombosis either during surgery or during the initial 24-hour postoperative period [17]. 

### 2.4. Health-Related Quality of Life

HRQoL was assessed using two validated quality of life (QoL) instruments that have been used in previous EVAR studies [18–26]. The Short Form-36 Health Survey (SF-36), a generic quality of life questionnaire [27], includes a multi-item scale that assesses eight health domains: physical functioning, social functioning, role-physical, bodily pain, general mental health, role-emotional, vitality, and general health perceptions. A score ranging from 0 to 100 can be generated for each domain with higher scores indicating a better quality of life. The European Quality of Life questionnaire (EQ-5D), a preference-based questionnaire, consists of five questions defining five health states in terms of mobility, self-care, usual activities, pain/discomfort, and anxiety/depression. For each of the five individual questions of the EQ-5D instrument, three degrees of impairment are possible: no impairment, some impairment, and extreme impairment. The individual responses can be transformed into a utility score on a 0-1 scale in which 0 corresponds to death and 1 to a perfect health state) [28]. 

### 2.5. Cost and Cost-Effectiveness Analyses

An economic evaluation of the trial data was conducted to compare the surgical options in terms of one-year expected costs and life years. To represent both payer and societal perspectives, resource utilization (e.g., hospitalization, physician visits) and productivity lost (e.g., days missed from work if employed) data were prospectively collected. LHSC case costing data and public sources were used to cost out the resource utilization and productivity losses. For each treatment group, life-years were determined and used as the primary effectiveness measure for the cost-effectiveness analysis. In the absence of dominance (e.g., EVAR more effective and less costly than OSR), the incremental cost per life-year gained of EVAR compared to OSR was determined for low and high risk OSR patients. All cost values in the paper are expressed in 2006 Canadian dollars (CAD).

### 2.6. Statistical Analyses

Continuous and discrete variables were summarized using mean values (standard deviations) and percentages, respectively. Statistical significance was conducted using Chi-square tests for categorical variables and *t-tests* for continuous variables. Kaplan Meyer curves were used to compare-treatment options and determine life years over the one year period following the surgery. In the economic analysis, one-year costs and effects were bootstrapped to express sampling uncertainty associated with the trial data. The analyses were conducted for EVAR, OSR among patients at a low surgical risk (OSR LR) and high surgical risk (OSR HR). 

## 3. Results

### 3.1. Patient Baseline Characteristics

Between August 11, 2003 and April 3, 2005, 140 patients were treated with EVAR, 195 with OSR, and 7 with BMT. Of the patients undergoing OSR, 52 patients were high risk and 143 low risk. As shown in Table 1, EVAR- and OSR-HR treated patients were comparable with respect to their baseline characteristics with the exception of gender (*P* = .04) and the presence of peripheral vascular disease (*P* < .01). In contrast, the OSR LR group was younger and had fewer baseline comorbidities (Table 1). 

### 3.2. Primary Technical Success

Among EVAR patients, the PTS was 100%. All EVAR patients had successful introduction and deployment of the endografts during the procedure, and there were no surgical conversions, deaths, type I and III endoleaks, or graft limb obstructions. Although almost 50% (47.9%) of type II endoleaks were reported on the completion angiogram at the time of the initial procedure, these type II endoleaks did not require an immediate corrective procedure and the majority remained of no clinical significance over the study followup. PTS was assisted by the completion of three unplanned endovascular procedures in 2.1% of the EVAR patients. Four EVAR patients (2.9%) had to undergo a planned endovascular procedure and two others (1.4%) had additional procedures (e.g., inguinal hernia repair). For OSR patients, PTS was also 100%. 

### 3.3. Short and Mid-Term Mortality

**Figure 2:**
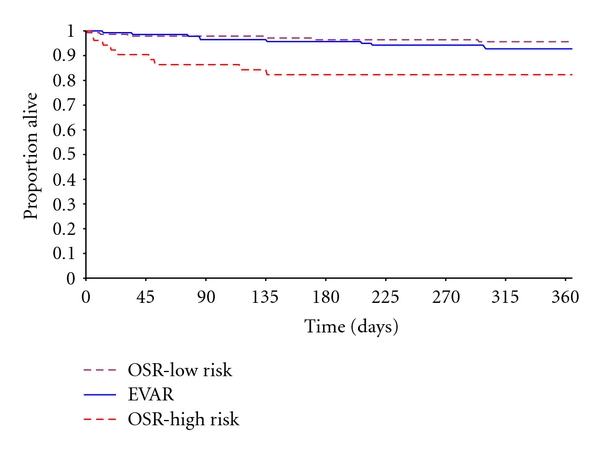
Kaplan Meier Survival Plots for EVAR, OSR HR, and LR patients up to 12 Months of Followup Kaplan Meyer.

The 30-day mortality rate associated with EVAR (1 patient, 0.7%) was significantly lower than the OSR HR group (5 patients, 9.6%, *P* < .01) and similar to the OSR LR group (2 patients, 1.4%, *P* = 1.0). The one postoperative death observed in the EVAR group was attributed to cardiac complications. Deaths in the OSR group were due to cardiac complications, pneumonia, respiratory failure, or multiorgan failure. The one-year mortality rate associated with EVAR was significantly lower compared to OSR HR patients (7.1% versus 17.3% resp.; *P* = .04) and comparable to OSR LR patients (7.1% versus 4.2% resp.; *P* = .28). The Kaplan Meier survival curves for the 3 patient groups are presented in Figure 2. Out of a maximum of 1 in our one-year study, the life years associated with EVAR, OSR LR, and OSR HR were calculated at 0.96 (SD: 0.15), 0.97 (SD: 0.15), and 0.85 (SD: 0.34), respectively. Out of 140 patients enrolled in EVAR, two were lost over the 5-year followup period. With 50 deaths among the 138 EVAR patients followed up for 5 years, the five-year mortality rate was 36%. The main reasons of death were cancer (*N* = 10), cardiac (*N* = 8), respiratory (*N* = 4), renal diseases (*N* = 4), other (*N* = 3), and unknown (*N* = 21). 

### 3.4. Other Perioperative and Postoperative Complications

Complications at the time of surgery occurred in 2.7% of all EVAR and OSR patients. No procedural graft thrombosis, vein, or nerve injuries were observed. The percentage of patients receiving blood transfusion at time of surgery was statistically lower in EVAR patients (1%) than in OSR LR (19.6%; *P* < .01) and HR (46.2%; *P* < .001) patients. The need for additional procedures at time of surgery (e.g., renal artery revascularization and inferior mesenteric artery reimplantation) was not statistically significantly different between EVAR and OSR patients.

The 30-day postoperative complication rates were in general lower in the EVAR patients compared to the OSR HR patients and similar to OSR LR patients (Table 2). Although 8.6% of EVAR patients had new type II endoleaks within one year, they did not require any reintervention nor were there any cases of aneurysm rupture, graft migration, integrity problems, or obstruction of the endografts. One-year vascular reoperation rates between treatment groups were not statistically different (i.e., 0.7% for EVAR, 2.1% for OSR LR, and 4.4% for OSR HR). 

### 3.5. Health-Related Quality of Life

**Figure 3:**
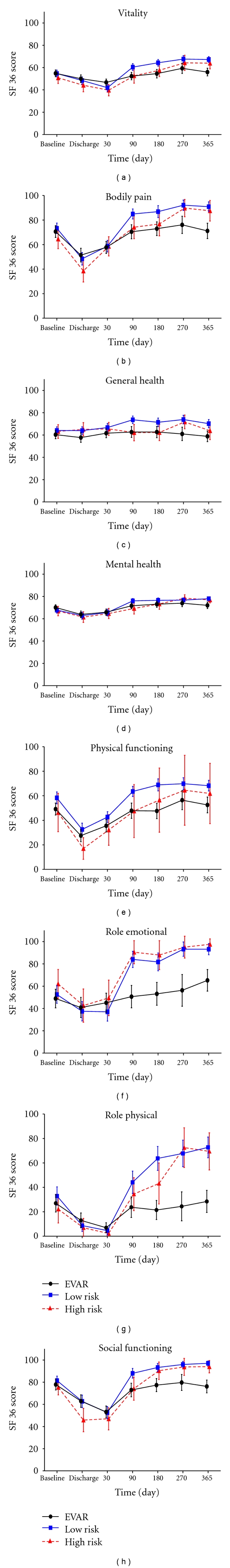
SF-36 domain scores and confidence intervals.

**Figure 4:**
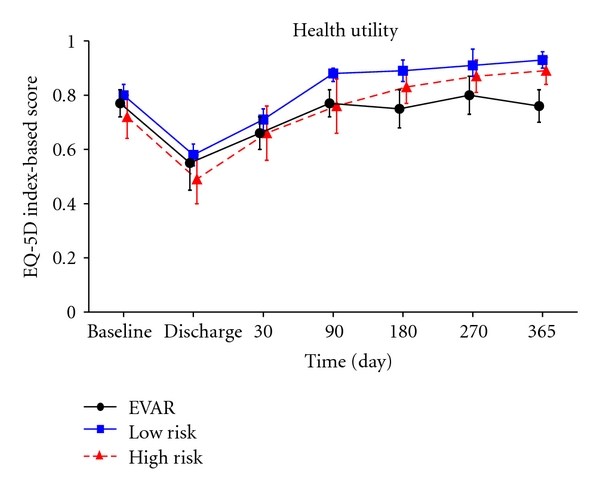
EQ-5D utility scores and confidence intervals.

No statistical differences were observed in the baseline values of the eight dimensions of the SF-36 between the treatment groups (Figure 3). Five-dimension scores (bodily pain, social functioning, role physician, role emotional, and physical functioning) dropped following the operation before returning to preoperative levels after 3 months for OSR LR and HR patients. In contrast, for EVAR patients, the scores associated with four of these five domains returned to the preoperative levels after 6 months and after 12 months for the fifth domain (role physical). At one year, EVAR patients had statistically lower scores compared to OSR HR or OSR LR patients for these five dimensions of the SF-36. No major changes were observed over time and between treatments for the three other domains of the SF-36 (general health, mental health, and vitality) (Figure 4). 

Similar to the pattern observed with the SF-36 data, patients' utilities decreased following the surgery and increased over time thereafter (Figure 4). The utility scores at 1 year were higher than the preoperative values for all OSR patients. EVAR patients returned to their perioperative levels. While the EQ-5D utility scores were similar at baseline between the three groups, the 12-month utility scores were lower for EVAR patients at 0.76 (SD: 0.26) compared to 0.89 (SD: 0.12) for OSR LR (*P* < .001) and 0.93 (SD: 0.12) for OSR LR (*P* = .015). Similar to the SF-36 results, the 1-year scores were not different between OSR patients (i.e., LR and HR groups). 

### 3.6. Costs and Cost Effectiveness

From a resource utilization point of view, EVAR required less procedural time (162.4 minutes ± 46.5) than OSR in LR patients (181 minutes ± 52.2) and in HR patients (195.8 minutes ± 64.8) (*P* <  .01 for both comparisons). In our study, 95% of all EVAR patients received general anesthesia while the majority of OSR patients received a general anesthesia with epidural (67.3% for OSR HR and 76.9% for OSR LR). Compared to OSR, EVAR patients spent less time in the hospital and required fewer admissions to the intensive care unit (ICU) (Table 3). Despite the additional cost of the graft (e.g., approximately $10,000), the mean initial costs of hospitalization were similar between the EVAR and OSR HR groups ($28,139 versus $31,181, *P* = .28 resp.). Costs of hospitalizations associated with OSR LR patients were statistically significantly less at $15,494 versus EVAR,   *P* <  .05 (Table 3). 

As the use of healthcare resources in the year following the operation was higher in EVAR patients, the average 1-year medical cost of followup was significantly higher for EVAR patients ($5,172) than OSR HR patients ($2,171) or OSR LR patients ($1,890) (Table 3). No statistical differences were observed in terms of indirect costs between the three treatment groups (Table 3). In total, the average 1-year cost of EVAR was $34,146 per patient, which compared to $34,170 per OSR HR patient and $19,163 per OSR LR group. The costs associated with OSR among LR patients were statistically lower compared to the other two groups (*P* < .05 for both comparisons). 

**Figure 5:**
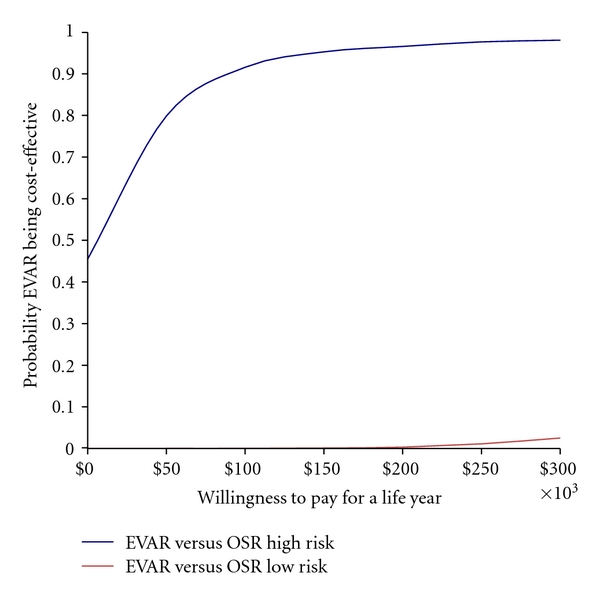
Cost-effectiveness acceptability curves ($/LYG).

Point estimates indicated that EVAR was less costly ($−24) and offered additional benefits (i.e., 0.12 life-year gained) than OSR in high risk patients. However, EVAR was dominated by OSR in low risk patients (e.g., OSR less costly and more effective than EVAR in high risk population). These results held true even when the uncertainty was taken into account as shown in Figure 5 which presents the cost-effectiveness acceptability curves used to represent the uncertainty. The way to interpret these curves is to consider a threshold that decision makers might be willing to pay for a unit of effect (i.e., willingness to pay per one life-year gained) along the horizontal axis and read along the vertical axis the probability that the treatment is cost-effective after accounting for uncertainty. For example, if society is willing to pay an extra $50,000 and $100,000 to save one extra year of life, the probability of EVAR being cost-effective compared to OSR in high risk patients was 0.76 and 0.90, respectively, while these probabilities were almost 0 when EVAR was compared to OSR in low risk patients. 

## 4. Discussion

The results of this 1-year study showed that at the current price of the endografts (approximately $10,000 at time of study), EVAR in high risk patients may be cost-effective compared to OSR in high risk patients but not in low risk patients. Specifically, a significant reduction in the 30-day and 1-year mortality rates was observed in favor of EVAR for high risk patients. However, when EVAR in high risk patients was compared to OSR in low risk patients, these mortality differences were not significant. In terms of costs, the 1-year cost of EVAR was similar to OSR in high risk patients despite the additional cost of the graft. In comparison, treating low risk patients with OSR was estimated to save almost $15,000 per year for similar mortality benefits. In addition, the study consistently showed that at 1-year OSR patients, independent of their surgical risk, had a better HRQoL than EVAR patients. This study also found that scoring algorithms could be used to identify patients who would benefit the most from EVAR (e.g., patients at a high risk of perioperative morbidity and death) or OSR (e.g., low risk patients). This was confirmed in our study as EVAR and OSR high risk patients were similar with respect to baseline variables (e.g., age, AAA size, Leiden mortality rates) despite the nonrandomized nature of the trial. In comparison, low risk patients undergoing OSR were younger and had fewer comorbidities than high risk patients treated with EVAR and OSR. 

Our study is unique in several ways. As opposed to the randomized trials EVAR, DREAM, ACE, and OVER which enrolled patients with a low to moderate surgical risk [19, 24, 29, 30], all EVAR patients were high risk patients in our study. In comparison approximately 7% of EVAR and OSR patients enrolled in the OVER trial were at high surgical risk, while more than half of the patients were at low risk. Similarly many nonrandomized studies have included a mixed patient risk populations [31]. As such it is difficult to compare our results with all previously completed randomized or nonrandomized studies. In contrast to other studies, we also present outcome data on low and high risk patients treated with OSR thus providing previously unavailable information. Finally we collected health-related quality of life and resource utilization data to perform an economic evaluation of the trial, which was used to inform policy making in Ontario. As such our data are very relevant from a clinical practice point of view as it is aligned with the current Canadian guidelines for the management of AAAs (e.g., EVAR not recommended in low risk patients) and funding recommendations in Ontario (EVAR not reimbursed for low risk patients). 

It is also difficult to compare our cost-effectiveness findings with previous studies. With the exception of a few studies, EVAR has not been found cost-effective compared to OSR which may be due to the fact that many randomized or observational studies do not explicitly separate out OSR patients based on presurgical risk. For example, our own 10-year decision analytic model populated with clinical data derived from a systematic literature and cost data from the field evaluation indicated that at an incremental cost of more than $400,000 per life year gained, EVAR was not cost-effective compared to OSR in Canada [12]. In this paper, the 30-day mortality rate derived from a systematic literature review was 1.5% for EVAR (compared to 0.7% in our study) and 4% for OSR (compared to 1.4% in low risk patients and 9.6% in high risk patients receiving OSR). When the trial 30-day mortality rates observed in high risk patients who underwent EVAR and OSR were used in the model decision model (i.e., a difference of 8.2% in 30-day mortality versus 2.5% in the original publication), EVAR became cost-effective at a cost-effectiveness ratio of less than $50,000 per LYG over a 10-year time horizon. Recent model-based cost-effectiveness analyses have also shown that EVAR may be cost-effective in high risk patients. For example, the model conducted by NICE to inform the use of EVAR in the UK concluded that “the ICER for patients of good fitness suggested that EVAR was not what would be usually agreed as a good use of NHS resources in these patients” [6, Section  4.3.14, page 27]. However, in the absence of “nationally agreed definitions of fitness for surgery”, the Committee felt that it would be inappropriate to exclude a specific group of patients suitable for surgery. NICE however recognized that the decision between EVAR and OSR should be individualized based on AAA size, patients' morphology and fitness to OSR [5]. In our study, low risk patients were treated with OSR and high risk patients anatomically suitable for EVAR were offered both surgical options. Results indicated that OSR was the most attractive option for low risk patients from a health economic standpoint. Although EVAR is a clinically acceptable and effective procedure for low risk patients, currently it is more expensive. Finally, our 5-year mortality rate of EVAR at 36% was comparable to 6-year mortality rate of 31% that was reported by DREAM trial [32]. Since OSR patients did not require similar long-term followup, we were not able to provide any information regarding their 5-year mortality. 

Several limitations were associated with our study including its nonrandomized nature. However, high risk patients treated with EVAR and OSR were similar in terms of baseline characteristics allowing direct comparisons between these two populations of HR patients. Although OSR patients at a lower surgical risk were not comparable with EVAR or OSR HR patients, we did not adjust the results comparing EVAR HR patients and OSR in LR patients, which may be a limitation of the analyses comparing EVAR versus OSR in LR patients. However, it is expected that OSR patients at a lower surgical risk will spend less time in the hospital that their high risk counterparts. It was therefore not surprising that the difference of $15,000 observed in the 1-year costs between OSR HR and LR patients was mostly due to the higher costs of hospitalization observed in the high risk group. Interestingly and despite the observational nature of our data, the three treatment groups were similar in terms of HRQoL prior to the surgery (e.g., SF-36 domains and EQ-5D utilities). Another limitation associated with this study was related to the limited number of OSR high risk patients (*N* = 52). However, bootstrap techniques were used to deal with the uncertainty associated with the trial data, and CEACs were used to represent this uncertainty. This study was also conducted in a single hospital in Ontario which may limit the generalizability of the results. It is also important to note that LHSC, which has an established endovascular program, is a primary center of referral and may not reflect the mix of patients seen in other institutions. Finally all enrolled patients had an AAA > 5.5 cm and as such the results cannot be generalized for smaller aneurysms. 

 Despite these limitations, this study provides new and currently unavailable information on the comparative costs and outcomes associated with treatment options for patients with an AAA > 5.5 cm. Our results do not support reimbursement of EVAR for low risk patients at the current price of the endografts despite a similar clinical effectiveness. In light of these findings and to better inform decision making around EVAR, future trials of EVAR should use scoring algorithms to allow stratified analyses by surgical risk.

## Figures and Tables

**Table 1:** Baseline characteristics of study participants.

Variable	EVAR	OSR high risk	OSR low risk	*P* Value EVAR versus OSR high risk	*P* Value EVAR versus OSR high risk	*P* Value OSR high risk versus low risk
No. of patients, %	140 (100)	52 (26.6)	143 (73.4)			
Age, yr (mean, SD)	75.6 ± 7.8	74.0 ± 7.9	71.7 ± 7.9	.24	<.01	.06
min-max	59–93	54–91	52–87			
Male gender, %	85.7	73.1	87.4	.04	0.68	.02
*Smoking, %*				.07	<.01	.53
Current	22.8	34.6	40.6			
Former	63.6	61.5	53.1			
Never	13.6	3.9	6.3			
Mean AAA size (cm)	6.2 ± 0.9	6.5 ± 1.0	5.9 ± 1.0	.10	0.98	.10
SVS/ISCVS risk-factor scores, %				.97	<.01	<.01
I	34.3	34.6	83.9			
II	65.7	65.4	16.1			
ASA Grade, %				.69	<.01	.02
I	0	0	0			
II	1.4	0	4.2			
III	32.1	33.3	50.3			
IV	66.5	66.7	45.5			
Leiden score (% of mortality)	6.9 ± 4.3	7.2 ± 10.0	4.1 ± 2.8	.76	<.01	.04
Cardiovascular history, %	35.7	42.3	19.0	.40	<.01	< .01
Angina pectoris MI						
<6 months	2.1	3.9	0.7	.61	.37	.17
>6 months	43.9	40.4	23.8	.66	<.01	.02
CHF, %	9.3	9.6	0	.94	<.01	<.01
Arrhythmia	25.0	21.2	7.8	.58	<0.01	<.01
Previous cardiac intervention angioplasty/stent						
GABG	11.4	7.7	7.0	.45	.20	1
Valve surgery	26.4	23.1	12.6	.64	<.01	.07
Hypertension	3.6	0	1.4	.33	.28	1
Stroke	81.3	75.0	74.1	.34	.15	.90
TIA	12.9	5.8	4.2	.16	<.01	.70
PVD	7.9	11.5	6.3	.41	.61	.23
	10.1	30.0	8.6	<.01	.67	<.01
DM	19.3	19.2	11.9	.99	.09	.19
Renal disease, %	1.4	0	0.7	1	1	1
Pulmonary disease, %						
COPD, %	35.7	41.2	20.4	.49	<.01	<.01
Emphysema, %	25.0	17.3	8.1	.26	<.01	.11
Asthma, %	2.9	7.7	4.9	.22	.38	.49
Other, %						
Previous abdominal surgery	45.3	34.6	28.7	.18	<.01	.48
Hostile abdomen	2.2	3.9	0.0	.61	.12	.07

AAA: Abdominal aortic aneurysm; ASA: American Society of Anesthesiologist; CHF: congestive heart failure; CABG: coronary artery bypass grafting; COPD: chronic pulmonary disease; EVAR: endovascular aneurysm repair; MI: myocardial infarction; OSR: open surgical repair; SD: standard deviation; SVS/ISVS: Society for Vascular Surgery/International Society for Cardiovascular Surgery.

**Table 2:** Postoperative complications (30 days or to discharge).

Postoperative complications	EVAR (*n* = 140)	OSR High Risk (*n* = 52)	OSR Low Risk (*n* = 143)	EVAR versus OSR High Risk	EVAR versus OSRLow Risk	OSR High versus Low Risk
Death	1 (0.7%)	5 (9.6%)	2 (1.4%)	*P* < .01	*P* = 1.00	*P* = .02
MI	6 (4.3%)	5 (9.6%)	7 (4.9%)	*P* = .17	*P* = .80	*P* = .31
CHF/Pulmonary edema	5 (3.4%)	9 (17.3%)	16 (11.1%)	*P* < .01	*P* = .49	*P* = .26
Arrythmia	5 (3.6%)	5 (9.6%)	8 (5.6%)	*P* = .14	*P* = .40	*P* = .34
Stroke	1 (0.7%)	0	0	*P* = 1.00	*P* = 1.00	*P* = 1.00
Renal failure	5 (3.6%)	6 (11.5%)	5 (3.5%)	*P* = .07	*P* = 1.00	*P* = .07
Pneumonia	0	4 (7.7%)	7 (4.9%)	*P* < .01	*P* = .02	*P* = .04
Sepsis	0	3 (5.8%)	1 (0.7%)	*P* = .02	*P* = 1.00	*P* = .06
Paralytic ileus	0	4 (7.7%)	6 (4.2%)	*P* < .01	*P* = .03	*P* = .46
Wound Infection/lymphocele	1 (0.7%)	1 (1.9%)	0	*P* = .27	*P* = .50	*P* = .27
Graft occlusion	0	0	0	*P* = 1.00	*P* = 1.00	*P* = 1.00
Blood transfusion	11 (7.9%)	11 (21.2%)	8 (5.6%)	*P* = .02	*P* = .45	*P* < .01
Vascular reoperation	1 (0.7%)	1(4.4%)	3 (2.1%)	*P* = .50	*P* = .62	*P* = 1.0
Arterial embolus	1 (0.7%)	0	1 (0.7%)	*P* = 1.00	*P* = 1.00	*P* = 1.0
Urinary tract infection	0	1 (1.9%)	1 (0.7%)	*P* = .27	*P* = 1.00	*P* = .46
GI bleed	0	0	2 (1.4%)	*P* = 1.00	*P* = .50	*P* = 1.00
Other surgery	1 (0.7%)	3 (5.77%)	1 (0.7%)	*P* = .06	*P* = 1.00	*P* = .66

**Table 3:** One-year resource utilization and cost.

Variable	EVAR	OSR, High risk	OSR, Low risk	Difference EVAR versus OSR High risk	Difference EVAR versus OSR Low risk	Difference OSR High—versus Low risk
*No. of patients*	140	52	143			
*Initial hospitalization*						
Duration of stay in hospital, day (mean, SD)	7.7 (5.8)	16.1 (16.0)	9.4 (5.2)	−8.43	−1.66	6.77
Frequency of attendance in ICU, %	5 (3.6)	16 (30.8)	9 (6.3)	−27.20	−2.70	24.5
Mean duration of stay in ICU (SD)	0.23 (1.71)	3.21 (8.25)	0.27 (1.4)	−2.98	−0.04	2.94
*Subtotal costs*	$28,139	$31,181	$15,494	−$3,042	$12,645*	−$15,687*
*Followup, mean number of: *						
Hospital admissions	0.36	0.13	0.20	0.23	0.16	−0.07
ER Visits	0.93	0.31	0.51	0.62	0.42	−0.20
GP Visits	6.37	5.48	5.84	0.89	0.54	−0.35
Specialist visits	4.19	1.65	1.65	2.54	1.85	−0.69
Vascular surgeon visits	2.74	0.96	0.96	1.78	1.47	−0.31
CT scans	2.61	0.10	0.10	2.51	2.55	−0.04
*Subtotal costs *	$5,172	$2,171	$1,890	$3,010*	$3,282*	$272
*Followup, productivity*						
Mean paid days taken off work	3.91	3.23	8.90	0.68	−4.99	−5.67
Mean hours of care provided by others	18.6	23.0	35.07	−4.40	−16.47	−12.07
*Subtotal costs*	$835	$818	$1,779	$17	−$944	−$961
Total costs	34,146	$34,170	$19,163	−$24	$14,983*	−$15,007*

*: Indicate significance at 5% level.
